# Prokaryotic Community Composition in Arctic Kongsfjorden and Sub-Arctic Northern Bering Sea Sediments As Revealed by 454 Pyrosequencing

**DOI:** 10.3389/fmicb.2017.02498

**Published:** 2017-12-12

**Authors:** Yin-Xin Zeng, Yong Yu, Hui-Rong Li, Wei Luo

**Affiliations:** Key Laboratory for Polar Science of State Oceanic Administration, Polar Research Institute of China, Shanghai, China

**Keywords:** diversity, prokaryotic community, sediment, northern Bering Sea, Kongsfjorden

## Abstract

Fjords and continental shelves represent distinct marine ecosystems in the pan-arctic region. Kongsfjorden is a glacial fjord that is located on the west coast of Svalbard, and is influenced by both Atlantic and Arctic water masses. The Bering Sea consists of a huge continental shelf in the northeast and a deep ocean basin in the southwest, and is influenced by Pacific water. Microbial community compositions of Arctic sediment samples BJ4 from outer basin and BJ36 from inner basin of Kongsfjorden and sub-Arctic samples NEC5 from shallow shelf and DBS1 from deep basin region of the northern Bering Sea were investigated using 454 pyrosequencing of archaeal and bacterial 16S rRNA genes. Most archaeal sequences in the sediments were related to *Thaumarchaeota*, though *Euryarchaeota* were more abundant in the Arctic glacier-influencing inner basin sediment BJ36. *Thaumarchaeota* Group C3 was the dominant archaeal population in all samples. *Proteobacteria* and *Bacteroidetes* dominated the sediment bacterial communities. *Acidobacteria* and *Actinobacteria* were also dominant in the northern Bering Sea samples. *Alphaproteobacteria* and *Epsilonproteobacteria* were the two main classes in Kongsfjorden sediment bacterial communities while *Deltaproteobacteria* and *Gammaproteobacteria* were dominant in the northern Bering Sea sediments. Differences in the presence and abundance of other dominant archaeal and bacterial populations were observed among sediment samples. In contrast to archaeal community differences that the Arctic BJ36 archaeal community was distinct from the sub-Arctic sediments and the Arctic outer basin sediment BJ4, cluster analysis based on bacterial OTU (operational taxonomic unit) distributions indicated that the Arctic and sub-Arctic bacterial communities segregated from one another. These results suggest that the sediment archaeal and bacterial community compositions can be driven by different environmental factors. Differences in the presence and abundance of particular archaeal species (e.g., *Candidatus* Nitrosopumilus and *Methanococcoides*) or bacterial species (e.g., *Sulfurimonas, Sulfurovum*, and *Desulfobulbaceae*) involved in biogeochemical cycles were also observed among sediment samples. At the same time, despite the community variation, some phylotypes (e.g., *Marinicella*) were dominant in all sediments. This study indicates diverse microbial communities inhabiting pan-arctic marine sediments, and highlights potential roles for *Archaea* and *Bacteria* in global biogeochemical cycles in these environments.

## Introduction

Microorganisms exist in marine sediments in high abundances (>10^8^ cells per gram; Cammen, 1982; Montagna, 1982; Schmidt et al., 1998; Danovaro et al., 2002), and possess a variety of catabolic enzymes that allow them to decompose various substrates efficiently (D’Hondt et al., 2002). As a result, marine sediment microbial communities have profound impacts on global biogeochemical cycles (Jorgensen et al., 2012), and are also important members of the marine food web structure (Epstein, 1997). Microbial communities of marine sediments are generally dominated by a restricted number of bacterial and archaeal phyla, including *Chloroflexi, Planctomycetes*, Japanese Sea division 1 (JS-1), diverse *Proteobacteria*, the Deep Sea Archaeal Group (DSAG), Marine Group I (MG-I) *Archaea*, the Miscellaneous Crenarchaeotic Group (MCG), and the South African Goldmine Euryarchaeotal Group (SAGMEG) (Fry et al., 2008; Teske and Sørensen, 2008; Orcutt et al., 2011). At the same time, marine sediment communities are impacted by numerous chemical and physical parameters (Jorgensen et al., 2012; Liu et al., 2015; Nguyen and Landfald, 2015), and these communities can be sensitive to environmental changes. Microbial community compositions in marine sediments also can be affected by geographic distance and ocean current (Hamdan et al., 2013; Xiong et al., 2014). In keeping with ‘Everything is everywhere; but the environment selects’ (Baas-Becking, 1934), microorganisms are transported in oceanic masses over long distances, and local sediment features control recruitment from the water column (Hamdan et al., 2013). Therefore, it is supposed that microbial communities in different marine sediment environments contain both geographically cosmopolitan microorganisms and locally endemic taxa.

Arctic fjords and open continental shelves are two of distinct marine ecosystems in the pan-arctic region. Kongsfjorden is an Arctic glacial fjord that is located on the west coast of Spitsbergen in the Svalbard archipelago at 79°N, 12°E. It is influenced by intrusion of Atlantic water (AW) from the West Spitsbergen Current (WSC) and transports relatively warm saline water (temperature >3°C and salinity >34.9) northward (Svendsen et al., 2002; Cottier et al., 2005). Therefore, the fjord is characterized by relatively mild temperatures, compared with other Arctic locations at similar latitude (Piquet et al., 2014). At the same time, the fjord is fed with freshwater by several large tidal glaciers and streams (Svendsen et al., 2002; Cottier et al., 2005). Tidal glaciers at the head of the fjord discharge freshwater and suspended loads that create steep environmental gradients in salinity, temperature, and sedimentation rates (Weslawski et al., 2000) and cause reduced biomass and diversity in the benthic community in the inner fjord (Hop et al., 2002). The meltwater discharge affects a large area in the fjord, up to 45 km distance from the glacier front and up to 30 m depth (Hop et al., 2002, 2006; Svendsen et al., 2002) and leads to strong surface stratification during summer (Piquet et al., 2014). The significant variation in the bacterioplankton community composition of outer and inner fjord indicates strong and localized influence of glacial melt water in shaping the community structure (Sinha et al., 2017). The Bering Sea consists of a huge continental shelf in the northeast and a deep ocean basin in the southwest. The sub-Arctic northern Bering Sea is one of the most productive ocean regions in the world due to nutrient-rich Pacific waters overflowing the shallow shelf on its northward transit to the Arctic Ocean. Hydrographically, the northern Bering Sea is more closely connected to the Arctic Chukchi Sea to the north than it is to the southern Bering Sea (Grebmeier et al., 2006). It is supposed that variation of bacterial and archaeal communities in sediments can be observed between the two geographically distant regions.

Microbial community analyses in previous studies have only used conventional, low-throughput Sanger sequencing-based methods (e.g., 16S rRNA gene clone library analyses; Tian et al., 2009; Zeng et al., 2011). Those methods do not allow for the detection of many microbial taxa, especially rare taxa, which may serve important ecological functions (Tedersoo et al., 2010). In fact, rare but cosmopolitan microorganisms may serve as a reservoir of microorganisms that can initiate the development of new communities when environmental conditions change (Pedrós-Alió, 2006). Our understanding of sediment microbial communities in the pan-arctic regions remains incomplete. Recently, massively parallel pyrosequencing technologies, such as FLX 454 pyrosequencing, have been widely used to identify microbial taxa from various Arctic environments, including seawater and marine sediments (Jorgensen et al., 2012; Zeng et al., 2013; Kilias et al., 2014; Han et al., 2015; Li et al., 2015; Zhang et al., 2016). 454 pyrosequencing provides orders of magnitude more data compared with Sanger sequencing-based methods, thus resulting in a more complete census of the diversity of organisms in their natural habitats (Tedersoo et al., 2010). Here, 454 pyrosequencing of 16S rRNA genes was employed to obtain deeper insights into the sediment microbial communities in Kongsfjorden and the northern Bering Sea. The aim of this study was to explore whether sediment community variations could be detected between the two distinct marine ecosystems, and whether geographically cosmopolitan phylotypes could be found among sediments in the pan-arctic region. This study will enhance our knowledge of microbial life in Arctic and sub-Arctic marine sediments and provide a more comprehensive insight into the function of natural microbial communities in these environments.

## Materials and Methods

### Study Sites and Sediment Collection

**FIGURE 1:**
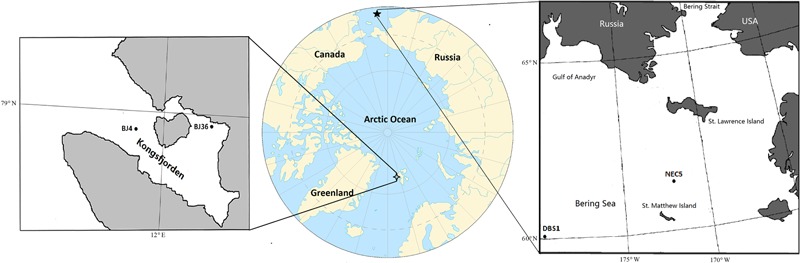
Map of sampling sites in Arctic Kongsfjorden and sub-Arctic northern Bering Sea.

The location and sampling date of sediment samples analyzed in this study are summarized in **Table 1** and **Figure 1**. The arctic Kongsfjorden is divided into two basins: the inner basin (glacial bays) with average depths of 40–60 m that is well-separated from the main fjord by a chain of islands, and the outer basin that has average depths of 200–300 m (Zhu et al., 2014). The inner basin hosts the Kongsbreen Glacier, which is the most active calving glacier in Svalbard. The glacial activity causes steep environmental gradients in salinity, temperature, and sedimentation rates (Svendsen et al., 2002). The inner basin sediments can be subject to strong disturbance due to the effects of glacial activity, while the outer basin sediments are exposed to relatively weaker disturbances due to greater seawater depths (Zhu et al., 2014). Sediment samples BJ4 from outer basin and BJ36 from inner basin of Kongsfjorden were collected using a van Veen Grab during the summer of 2008. Sub-Arctic sediment samples NEC5 from shallow continental shelf and DBS1 from deep basin region of the northern Bering Sea were collected between May and June in 2007 using a van Veen Grab (Zeng et al., 2011). Sampling site NEC5 is located on a continental shelf between St. Matthew and St. Lawrence Islands, where it is largely confined by continental land masses (Cooper et al., 2012). Most of the organic carbon produced over the northern Bering Sea actually remains on the shelf and is not transported to the deep basins (Piepenburg, 2005). Sediment samples were transferred to sterile plastic tubes and then stored at -80°C until DNA extraction.

**Table 1:** Characteristics of sediment samples analyzed for prokaryotic community composition.

Station name	Sampling date	Latitude	Longitude	Water depth (m)	Bottom water temperature (°C)^a^	Salinity (psu)^a^	pH^b^	Total carbon (mg g^-1^)^b^	Total nitrogen (mg g^-1^)^b^	Total sulfur (mg g^-1^)^b^	Total hydrogen (mg g^-1^)^b^	Total organic matter (mg g^-1^)^b^
BJ36	2008/7/30	78.984°N	12.376°E	28	–	–	8.0	15.1	0.49	0.54	0.16	9.0
BJ4	2008/7/28	78.960°N	11.921°E	350	–	–	8.2	31.8	1.12	2.06	0.30	16.3
NEC5	2007/5/18	61.389°N	171.951°W	62	-1.75	32.57	–	–	–	–	–	–
DBS1	2007/6/16	60.025°N	179.657°W	2420	2.1	34.61	–	–	–	–	–	–

*^a^Data was from Zeng et al. (2011).*

*^b^Data was from Zhu et al. (2014).*

### DNA Extraction, Amplification, and Pyrosequencing

Community DNA from each sediment was extracted from 2.0 g of sediment using an E.Z.N.A.^®^ Soil DNA Kit (OMEGA Bio-Tec, Inc., Norcross, GA, United States), according to the manufacturer’s instructions. Negative control was not used in the DNA extraction. Extracted DNA was visualized on a 1.0% agarose gel with ethidium bromide. The concentration of extracted DNA was determined using a NanoDrop 1000 spectrophotometer (Thermo Fisher Scientific, Inc., Wilmington, DE, United States). Results showed that the A260:A280 ratios were between 1.8 and 2.0 and that DNA concentrations were between 6 and 10 ng μl^-1^, indicating that the community genomic DNA met the requirements for subsequent sequencing.

To assess bacterial community composition, the V1–V3 regions of bacterial 16S rRNA genes were amplified using the universal forward primer 8F (5′-AGAGTTTGATCCTGGCTCAG-3′) with the Roche 454 ‘B’ pyrosequencing adapter (5′-CCTATCCCCTGTGTGCCTTGGCAGTCTCAG-3′) attached to the 5′-end of the primer, and the universal reverse primer 533R (5′-TTACCGCGGCTGCTGGCAC-3′) with the Roche 454 ‘A’ sequencing adapter (5′-CCATCTCATCCCTGCGTGTCTCCGACTCAG-3′) attached and a unique 8-bp barcode sequence at the 5′-end of the primer (Moreno et al., 2002; Wu et al., 2012). To assess archaeal community composition, the V3–V5 regions of archaeal 16S rRNA genes were amplified using the universal forward primer Arch344F (5′-ACGGGGYGCAGCAGGCGCGA-3′) with the Roche 454 ‘A’ sequencing adapter and a unique 8-bp barcode sequence attached, and the universal reverse primer Arch915R (5′-GTGCTCCCCCGCCAATTCCT-3′) with the Roche 454 ‘B’ pyrosequencing adapter attached at the 5′-end of each primer (Yu et al., 2008; Wu et al., 2012). Polymerase chain reactions (PCRs) were conducted using an ABI GeneAmp 9700 (Applied Biosystems, Foster City, CA, United States). The 20 μl PCR reaction mixtures contained template DNA (10 ng), 4 μl of 5× FastPfu buffer, 2 μl of 2.5 mM dNTPs, 0.4 μl of TransStart Fastpfu polymerase (AP221-02, TransGen Biotech, Co., Ltd., Beijing, China) and 0.4 μl of 5 μM of each primer. Positive (*Escherichia coli*) and negative controls (distilled water) accompanied reactions. The PCR program for bacterial PCRs consisted of an initial denaturation at 95°C for 2 min, followed by 25 cycles of denaturation at 95°C for 30 s, annealing at 55°C for 30 s, and extension at 72°C for 30 s, and a final extension at 72°C for 5 min. PCR amplification for archaeal PCRs consisted of an initial denaturation at 95°C for 2 min, followed by 37 cycles of denaturation at 95°C for 30 s, annealing at 53°C for 30 s, and extension at 72°C for 30 s, and a final extension at 72°C for 5 min. Amplicons from three separate PCRs were pooled for each sample.

Polymerase chain reactions products were extracted from 2.0% agarose gels and purified using an AxyPrep DNA Gel Extraction Kit (Axygen Biosciences, Corning, NY, United States) according to the manufacturer’s instructions. The DNA concentration of each sample was quantified using QuantiFluor^TM^-ST (Promega, Co., Madison, WI, United States). Amplicon pyrosequencing was performed from the ‘A’ end using a Roche 454 A sequencing primer kit on a Roche Genome Sequencer FLX Titanium Platform (Roche Applied Science, Indianapolis, IN, United States) at Majorbio Bio-Pharm Technology, Co., Ltd., Shanghai, China, following the vendor’s standard protocols. The raw sequence reads are deposited in the NCBI sequencing read archive under Accession No. SRP078034.

### Pyrosequencing Data Analyses

The Quantitative Insights into Microbial Ecology (QIIME) pipeline was employed to process the 16S rRNA gene pyrosequencing data, as previously described (Caporaso et al., 2010). Briefly, raw sequences without exact matches to the barcodes were discarded and the remainder were assigned to their respective samples and trimmed of primers and barcodes. Low-quality sequences were filtered based on the following criteria: length of <200 bp, the presence of ambiguous bases, average Phred score <25, and the presence of homopolymers of ≥6 bp length. Putative chimeric sequences were detected using USEARCH 6.1^1^ and removed from further analyses. The remaining high-quality sequences were clustered into operational taxonomic units (OTUs) at ≥97% nucleotide sequence identity using UPARSE-OTU algorithm (Edgar, 2013). Taxonomic ranks were assigned to representative sequences from each OTU using the Ribosomal Database Project (RDP) Naïve Bayesian Classifier v.2.2 (Wang et al., 2007) trained on the SILVA SSU database (SILVA version 115^2^), using a confidence value of 0.8 as the cutoff. Relative abundance of taxonomic groups within communities were then estimated from these data.

### Statistical Analyses and Microbial Community Characterization

Statistical analyses of 16S rRNA gene data were conducted as described by Wu et al. (2012). Alpha diversity indices, including the Chao 1, ACE, Shannon, and Simpson metrics were calculated in addition to Good’s coverage of diversity, and rarefaction analyses which were all assessed on OTU abundances using the Mothur software package^3^ (Schloss et al., 2009). The weighted UniFrac metric was computed for bacteria and archaea separately. Principal component analysis (PCA) was conducted using the genus-level classifications to evaluate compositional similarity among the microbial communities. A Venn diagram of shared and unique OTUs was generated using Mothur to visualize dissimilarity among microbial communities of the four sediments. Heatmap analysis was also performed to visualize OTU distributions and compare abundances across the four sediment samples. Shared taxa present in all four samples (100% threshold) were defined as the core microbiome.

## Results

### Sequence Analysis of Pyrosequencing Data

A total of 95,035 valid sequences were generated from four sediment samples. After quality filtering, 59,993 high-quality sequences (representing ∼63% of the total sequences) remained and were used in further analyses. Of these, 26,626 reads comprised the archaeal dataset and 33,367 reads comprised the bacterial dataset (**Table 2**). Average read lengths were 426 and 479 bp for *Archaea* and *Bacteria*, respectively. Singleton (containing only one sequence) and doubleton (containing two sequences) OTUs were removed from further analyses to more closely reflect the actual sediment community membership. Previous study indicates that removing low-frequency sequences is reasonable for reducing error rate and improving microbiota assessment (Allen et al., 2016). Good’s coverage estimation for *Archaea* in each sample was >99%. This result was consistent with rarefaction curve analyses (Supplementary Figure S1A), which indicated that the sequencing depth was sufficient to saturate the recovery of archaeal diversity in the sediment samples. Although bacterial rarefaction curves were not saturated after sampling 6,000–10,000 sequences in each sediment sample (Supplementary Figure S1B), Good’s coverage estimation for *Bacteria* in each sample was >86%, indicating that most of the bacterial diversity in the sediments was detected using our pyrosequencing approach. Diversity was generally higher for both *Archaea* and *Bacteria* in deep water sediments than in shallow water sediments located in the same geographic area. In addition, diversity was generally higher for *Bacteria* than *Archaea* in each sediment, which is consistent with previous studies on Alaskan Beaufort Shelf sediments (Hamdan et al., 2013). The 20 most abundant archaeal OTUs represented 86.2% of the total archaeal sequences. In contrast, the 20 most abundant bacterial OTUs accounted for just 32.1% of the total bacterial sequences.

**Table 2:** Comparison of phylotype coverage and diversity estimation of the 16S rRNA gene libraries from the pyrosequencing analysis.

Sample	Reads	OTU^∗^	Good’s coverage	Chao 1	ACE	Shannon	Simpson
*Archaea*							
BJ36	6,750	108	99.6	124	127	2.17	0.247
BJ4	6,304	159	99.7	162	168	3.2	0.114
NEC5	7,503	98	99.8	103	108	2.28	0.204
DBS1	6,069	127	99.7	130	134	3.01	0.092
*Bacteria*							
BJ36	10,487	1,156	93.7	2,057	2,723	4.66	0.051
BJ4	7,614	1,208	90.5	2,415	3,150	5.25	0.032
NEC5	6,868	1,555	86.7	2,741	3,860	6.12	0.007
DBS1	8,398	1,787	87.2	3,426	4,823	6.2	0.008

*^∗^The operational taxonomic units (OTUs) were defined at 97% sequence identity.*

### Archaeal Diversity and Community Structure

**FIGURE 2:**
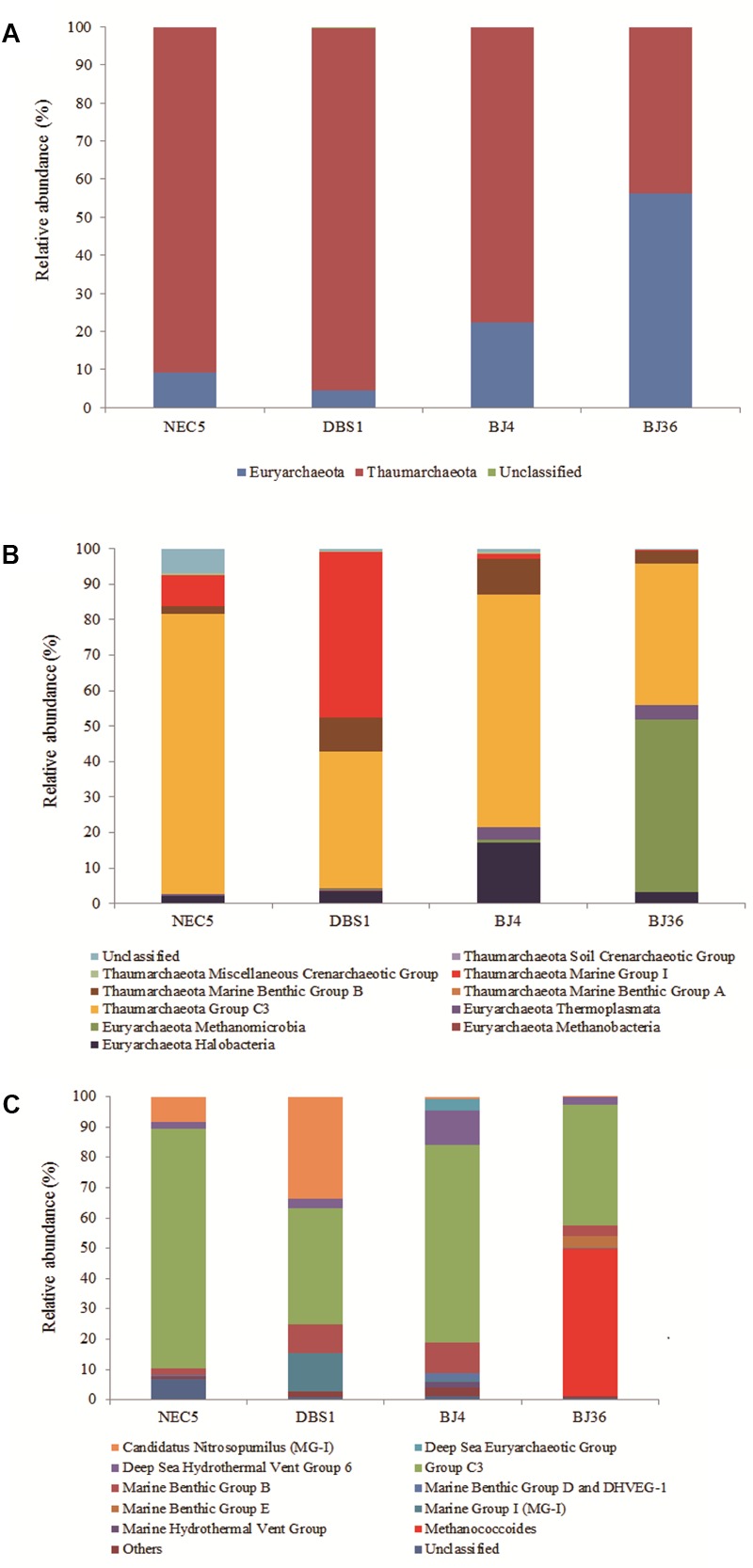
Distribution of *Archaea* at the phylum **(A)**, class **(B)**, and genus **(C)** levels in four sediment samples. Samples NEC5 and DBS1 were collected from the northern Bering Sea, whilst BJ4 and BJ36 were collected from Kongsfjorden, Svalbard. Others indicated the sum of genera which represented less than 1% of the total archaeal sequences in the four sediments.

A total of 281 archaeal OTUs were identified in this study. Besides taxonomically unaffiliated *Archaea*, two phyla were detected (**Figure 2A**). Archaeal distributions were characterized in terms of relative taxonomic abundances. *Archaea* that represented >1% of the relative abundance at different taxonomic levels were designated as dominant. *Thaumarchaeota* dominated archaeal community abundances and diversity, with the exception of sediment BJ36 where the *Euryarchaeota* were more abundant (56.2% of the total archaeal community). Over 76% of sediments archaeal sequences fell into the *Thaumarchaeota*.

At the class level, *Thaumarchaeota* Group C3 (38.4–79.1%) were the dominant archaeal taxa among all samples (**Figure 2B**). Of the other classes, the Euryarchaeota *Methanomicrobia* represented 48.7% of the archaeal sequences in Arctic sediment BJ36 while the Euryarchaeota *Halobacteria* and thaumarchaeota Marine Benthic Group B (MBG-B) represented 17.1 and 10.0% of the total archaeal sequences in Arctic sediment BJ4, respectively. MBG-B was the third-most abundant class (9.5%) in the sub-Arctic sediment DBS1. Thaumarchaeotal MG-I were dominant in sub-Arctic sediments, accounting for 8.7 and 46.6% of the archaeal sequences in samples NEC5 and DBS1, respectively. In addition, approximately 6% of the archaeal sequences in sediment NEC5 could not be classified beyond the phylum *Euryarchaeota* using the RDP classifier.

In addition to an unclassified group within the *Thaumarchaeota* Group C3 (represented by archaeal OTU97), the Euryarchaeota Deep Sea Hydrothermal Vent Group 6 (represented by OTU29) and an unidentified group of the thaumarchaeotal MBG-B (represented by OTU169) were dominant in all four sediments. Sequences affiliated with *Candidatus* Nitrosopumilus of the *Thaumarchaeota* (represented by OTU275) accounted for 8.5 and 33.7% of the total archaeal reads in the sub-Arctic samples NEC5 and DBS1, respectively, which contrasts with their lower abundance (less than 1% of the total archaeal sequences) in the Arctic sediments (**Figure 2C**). An unidentified archaeal group within the MG-I (represented by OTU75) was present in all sediments and was significantly more abundant (12.5%) in the deep water sediment DBS1. The Euryarchaeota *Methanococcoides* (represented by OTU234) and the Marine Benthic Group E (represented by OTU80) accounted for 48.7 and 3.8% of the *Archaea* in Arctic sample BJ36, respectively, while those archaeal groups were non-existent or of low abundance in other sediments.

**FIGURE 3:**
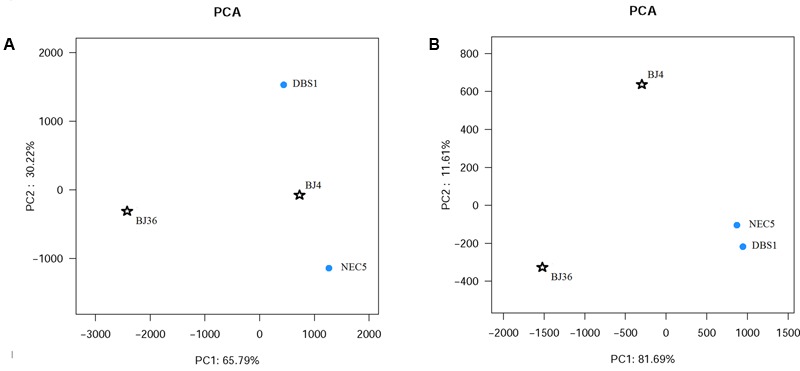
Principal component analysis (PCA) of the samples obtained by pyrosequencing and classified as **(A)** *Archaea* and **(B)** *Bacteria* using the UniFrac metric.

Principal component analysis (**Figure 3A**) based on archaeal OTU distributions indicated a clear separation between BJ36 and the other sediments, with the first principal component encompassing 65.7% of the total variation. Cluster analysis based on the 100 most abundant archaeal OTUs also indicated a similar result, with the Arctic shallow fjord sediment BJ36 comprising a separate branch from a group consisting of the sub-Arctic sediments and the Arctic deep fjord sediment BJ4 (Supplementary Figure S2). Taken together, the results suggest that the archaeal community structure in sediment BJ36 was distinct from the other three sediments.

### Bacterial Diversity and Community Structure

**FIGURE 4:**
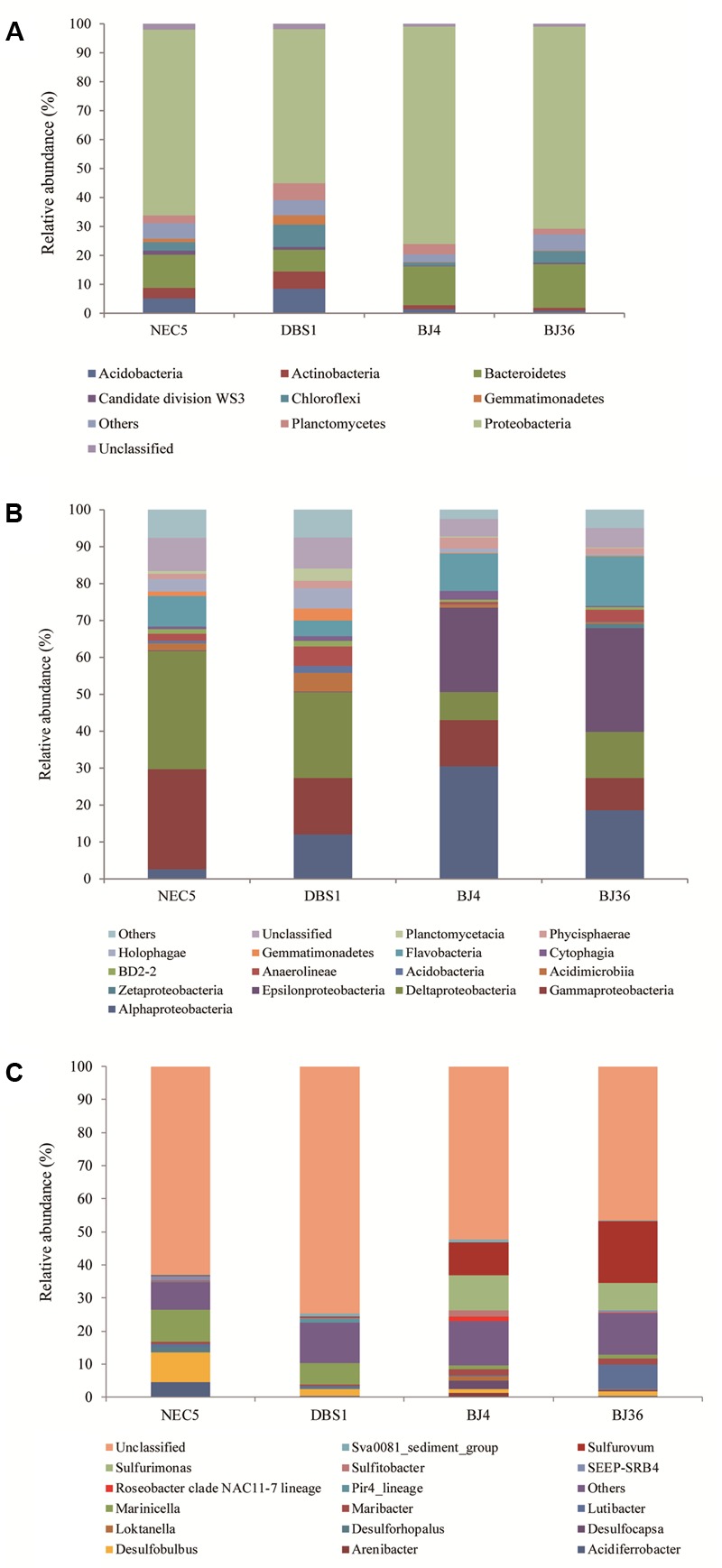
Distribution of *Bacteria* at the phylum **(A)**, class **(B)**, and genus **(C)** levels in four sediment samples. Samples NEC5 and DBS1 were collected from the northern Bering Sea, whilst BJ4 and BJ36 were collected from Kongsfjorden, Svalbard. Others indicated the sum of phyla, classes, or genera which represented less than 1% of the total bacterial sequences in the four sediments.

A total of 4,285 bacterial OTUs were identified in this study. Thirty-two bacterial phyla were detected in addition to other taxonomically unaffiliated bacteria. Bacteria taxa exhibiting a relative abundance greater than 1% of the total sequences at various taxonomic levels were designated as dominant. The seven most abundant bacterial phyla were *Proteobacteria, Bacteroidetes, Chloroflexi, Acidobacteria, Planctomycetes, Actinobacteria*, and *Gemmatimonadetes*, which together accounted for more than 93% of the total bacterial sequences. Sequences affiliated with *Acidobacteria, Actinobacteria*, and *Gemmatimonadetes* were more abundant in sub-Arctic sediments than Arctic sediments (**Figure 4A**).

*Proteobacteria* were the dominant phylum in all sediments and comprised more than 65% of all bacterial sequences. Among the *Proteobacteria*, the *Alpha*-, *Gamma*-, and *Delta*-*proteobacteria* were the dominant classes (**Figure 4B**). In addition, *Epsilonproteobacteria* were dominant in Arctic sediments BJ4 and BJ36, and accounted for 22.8–28.0% of the total sequences in each sediment. *Alphaproteobacteria* comprised a larger proportion (24.5% on average) of Arctic sediments compared to sub-Arctic sediments NEC5 and DBS1 (7.3% average). In contrast, *Gamma*- and *Delta*-*proteobacteria* constituted 21.2 and 27.5% of the sequences in sub-Arctic sediments, respectively, while they only comprised 10.6 and 10.0% of the sequences in Arctic sediments, respectively. The second-most abundant phylum in the four sediments was the *Bacteroidetes* which comprised 7.4–15.1% of the bacterial sequences in each sample. The *Flavobacteria*, followed by *Cytophagia* and BD2-2, were the most abundant classes of the *Bacteroidetes* and accounted for 4.2–13.4% of the total sequences in each sediment. The dominant class of *Chloroflexi* was the *Anaerolineae*, which represented 0.6–5.2% of the bacterial sequences in sediments. The most abundant class of the *Acidobacteria* phylum was the *Holophagae*, followed by the *Acidobacteria*, with each accounting for 0.4–5.5% of the total bacterial sequences in each sediment. The *Phycisphaerae*, followed by *Planctomycetacia*, were the most abundant classes of the *Planctomycetes* phylum and represented 1.4–3.0% of the bacterial sequences in sediments. The phylum *Actinobacteria* was dominated by bacteria belonging to the class *Acidimicrobia*, whereas the *Gemmatimonadetes* phylum was dominated by bacteria belonging to the class *Gemmatimonadetes*. Approximately 4.7–9.0% of the bacterial sequences could not be classified with confidence at the class level using the RDP classifier.

At the genus level, sequences affiliated with *Sulfurimonas* and *Sulfurovum* of the *Epsilonproteobacteria* class comprised a significant proportion (20.4–27.0%) of sediment *Bacteria* in the Arctic Kongsfjorden, while they were seldom detected in sediments of the sub-Arctic northern Bering Sea (**Figure 4C**). In addition, *Desulfocapsa* within the *Epsilonproteobacteria* accounted for 2.5% of *Bacteria* in the Kongsfjorden sediment BJ4. The genus *Lutibacter* within the *Bacteroidetes* comprised a substantial portion (7.4%) of the total bacterial sequences in the Kongsfjorden sediment BJ36. The genus *Desulfobulbus* (1.1–8.8%) within the *Deltaproteobacteria* and *Marinicella* (1.1–9.6%) within the *Gammaproteobacteria* were dominant in all sediments, but with much higher abundances observed in sub-Arctic sediments. Sequences affiliated with *Acidiferrobacter* within the *Gammaproteobacteria* comprised 4.5% of the bacterial sequences in the northern Bering Sea sediment NEC5.

Principal component analysis (**Figure 3B**) based on the distribution of bacterial OTUs exhibited a clear separation of bacterial community structure between Arctic sediments (BJ4 and BJ36) and sub-Arctic sediments (NEC5 and DBS1), with the first principal component representing 81.6% of the total variation. Cluster analysis based on the 100 most abundant bacterial OTUs also provided a similar result with the Arctic sediment communities comprising a cluster separate from the sub-Arctic sediment communities cluster (Supplementary Figure S3). These results indicated similarity in bacterial community structures of sediments from the same geographic location, but distinct bacterial community structure between sediments from different geographic locations.

### Core Microbiome

**FIGURE 5:**
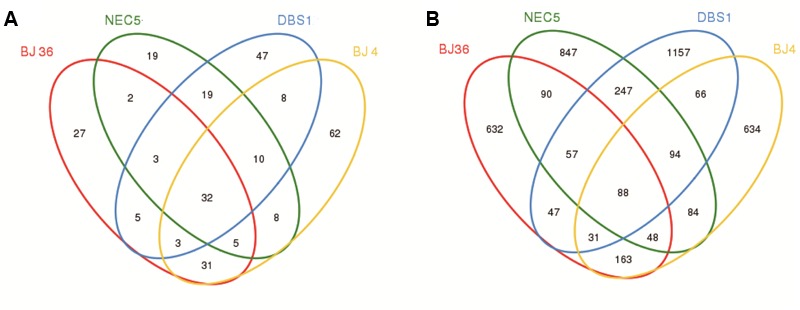
Venn diagrams showing shared and unique OTUs at 97% sequence identity for **(A)** *Archaea* and **(B)** *Bacteria* in four sediment samples.

To examine the existence of a common core microbiome among the four sediments, the core was defined as OTUs that were shared among all communities, as represented by overlapping areas in the Venn diagram analysis (Xiao et al., 2016). A total of 32 archaeal OTUs were shared among the four sediments (**Figure 5A**), which represented 11.3% of all archaeal OTUs (281 total archaeal OTUs) and 69.8% of total archaeal abundances. The ten most abundant archaeal OTUs that were shared belonged to the thaumarchaeotal Group C3 (73.7% of the core archaeal sequences), the MG-I group (7.8%), and the MBG-B group (6.0%). There were 62, 27, 19, and 47 unique archaeal OTUs observed in samples BJ4, BJ36, NEC5, and DBS1, respectively. The number of core archaeal OTUs exceeded the number of unique archaeal OTUs in the shallow water sediments NEC5 and BJ36. Further, most of the unique archaeal OTUs were also of low abundance. The unique OTUs can be considered as the variable microbiome in sediments. Some of the unique archaeal OTUs in Arctic Kongsfjorden sediments exhibited relatively high abundance. For example, the Marine Benthic Group E-related OTU80 and *Methanococcoides*-related OTU84 represented 3.3 and 3.0% of the total archaeal sequences in sediment BJ36, respectively. In contrast, the unique OTU167 that was affiliated with the Deep Sea Euryarchaeotic Group accounted for 1.2% of the *Archaea* in sediment BJ4.

A total of 88 bacterial OTUs were shared among the four sediments (**Figure 5B**), which accounted for only 2.0% of all bacterial OTUs (4,285 total bacterial OTUs), but 31.5% of bacterial abundances. The ten most abundant core bacterial OTUs were affiliated with the family *Rhodobacteraceae* of the *Alphaproteobacteria* (26.0% of the core bacterial sequences), the genus *Marinicella* and the JTB255 marine benthic group family of *Gammaproteobacteria* (8.1%), the genus *Desulfobulbus* and families *Sandaracinaceae* and Sva1033 of *Deltaproteobacteria* (9.3%), the genus *Sulfurovum* of *Epsilonproteobacteria* (18.4%), and the genus *Maribacter* and the family *Flavobacteriaceae* of the *Bacteroidetes* (3.4%). There were 634, 632, 847, and 1,157 unique bacterial OTUs found in samples BJ4, BJ36, NEC5, and DBS1, respectively. In contrast to the *Archaea*, the number of unique bacterial OTUs in all sediments exceeded the number of core bacterial OTUs. Most of the unique bacterial OTUs also exhibited low abundances. However, some of the unique OTUs were in relatively high abundance. In Kongsfjorden, the *Lutibacter*-related OTU1751, *Maritimimonas*-related OTU3746 and *Anaerolineaceae*-related OTU4059 represented 6.7, 1.3, and 1.5% of the total bacterial sequences in sediment BJ36, respectively, while the *Thiotrichaceae*-related OTU4062 accounted for 1.0% of all sediment bacteria in BJ4. In the sub-Arctic sediment DBS1, the unique OTU1753 affiliated with the family *Sandaracinaceae* and accounted for 5.9% of the total bacterial sequences in that sample.

## Discussion

A comprehensive and thorough investigation of microbial diversity in sediments is essential for understanding the ecological role of microorganisms in these habitats. The adoption of inexpensive and high-throughput pyrosequencing, and other next-generation sequencing techniques, now allows for the assessment of the full taxonomic diversity of microorganisms in marine sediments. Importantly, this approach can identify a large number of low-abundance or rare taxa in addition to predominant taxa. 454 FLX pyrosequencing was used in this study to improve our understanding of prokaryotic community structure in sediments of the Arctic Kongsfjorden and sub-Arctic northern Bering Sea. Twenty-one and 12 more phyla were found in the sediments of Kongsfjorden and the northern Bering Sea in this study, respectively, which contrasts with a total of 11 and 15 bacterial phyla detected using conventional clone library analysis in these samples (Tian et al., 2009; Zeng et al., 2011). In addition, our analyses indicated the presence of *Zetaproteobacteria* for the first time in Kongsfjorden sediments. 16S rRNA gene sequence related to the phylum *Chlamydia*e were not detected in this study, though one *Chlamydiae*-related sequence was previously detected in Kongsfjorden sediments (Tian et al., 2009).

In contrast to the predominance of *Gammaproteobacteria* in Kongsfjorden sediments in previous report (43.7% of the total bacterial sequences; Tian et al., 2009), *Alpha*- (24.5%), *Gamma-* (10.6%), *Delta-* (10.0%) and *Epsilonproteobacteria* (25.4%) were dominant in sediments analyzed here. In contrast, sequences affiliated with *Epsilonproteobacteria* were seldom detected in the northern Bering Sea sediments. The origin of water overlying sediments shapes benthic communities locally and globally and may dictate the recruitment of taxa from overlying waters (Hamdan et al., 2013). *Epsilonproteobacteria* were most abundant in Atlantic-influenced sediments on the Alaska Beaufort Shelf, and OTUs affiliated with the *Sulfurimonas* and *Sulfurovum* genera accounted for up to 13% of communities (Hamdan et al., 2013). In this study, the genera *Sulfurimonas* and *Sulfurovum* also represented a substantial portion of the sediment bacteria in Kongsfjorden, where an increased influx of AW into the Kongsfjorden system would influence sediment community composition from the outer to the inner fjord (Hop et al., 2002).

Differences in archaeal community structure were also observed between previous reports and this study. Tian et al. (2009) reported that the *Crenarchaeota* were the dominant taxonomic group in archaeal 16S rRNA gene clones, with only two sequences detected that were affiliated with the *Euryarchaeota*. However, in this study, the *Thaumarchaeota* (77.4% of total archaeal sequences) were dominant in the deep water sediment BJ4 while the *Euryarchaeota* (56.2%) were more abundant in the shallow water sediment BJ36. In the western Barents Sea sediments, the archaeal populations are largely (85.8%) affiliated with the *Thaumarchaeota* (Nguyen and Landfald, 2015). However, our assessment of bacterial community structure in the northern Bering Sea sediments was consistent with previous report that *Delta*- and *Gammaproteobacteria* were dominant in sediments of the northern Bering Sea (Zeng et al., 2011). The dominance of *Delta*- and *Gammaproteobacteria* has also been found in sediments from the Pacific Arctic Ocean (Li et al., 2009; Hamdan et al., 2013), which is influenced by both Pacific water via the Bering Strait and AW via the Fram Strait. This is the first report of archaeal community structure in sediments of the northern Bering Sea. Considering that the same northern Bering Sea sediment samples were used here as in a previous report (Zeng et al., 2011), the differences in microbial community composition of Kongsfjorden sediments between previous study (Tian et al., 2009) and this report can be attributed to variation in sediment samples rather than analytical methods.

The relationships between sediment microbial communities and environmental factors were not examined in this study for the absence of geochemical data for all four samples. However, despite documented variation in geochemical properties between Kongsfjorden sediments BJ4 and BJ36 (Zhu et al., 2014), sediment bacterial communities of the same geographic area were more similar to each other, but tended to exhibit more dissimilarity between spatially separated sediments (i.e., the northern Bering Sea) across large geographic distance. In comparison to the Arctic Kongsfjorden, *Acidobacteria, Actinobacteria*, and *Gemmatimonadetes* were more abundant in sub-Arctic northern Bering Sea sediments. In contrast, *Bacteroidetes* and *Proteobacteria* represented a more significant proportion of sediment bacteria in Kongsfjorden. However, with respect to sediment archaeal communities, shallow water sediments BJ4 from outer basin of Kongsfjorden and NEC5 from continental shelf of the northern Bering Sea were more similar to each other, regardless of their disparate geographic location. Results suggest differences in environmental factors shaping the sediment archaeal and bacterial communities. At the same time, both archaeal and bacterial communities in inner basin sediment BJ36 were distinct from those in outer basin sediment BJ4 that was from the same fjord in addition to two sub-Arctic sediments (**Figure 3**), which implicates an influence of glacial meltwater on sediment community composition.

Sequences affiliated with the *Zetaproteobacteria* were abundant (1.2% of the total bacterial sequences) in the shallow water sediment BJ36 in the inner basin, compared with the deep water sediment BJ4 in the outer basin of Kongsfjorden. All of the zetaproteobacterial OTUs in the present study belonged to the genus *Mariprofundus*, which are known as iron-oxidizing bacteria (Singer et al., 2011; Mumford et al., 2016; Makita et al., 2017). Growth of the genus *Mariprofundus* is oxygen dependent and requires marine salts (Emerson et al., 2007). Inner basin sediment BJ36 is close to the Kongsbreen Glacier and thus, is subject to strong disturbances due to effects of glacial activity. Glacial meltwater has been suggested to be a significant source of bioavailable iron to the oceans, in the form of both dissolved Fe and particulate Fe (Zhang et al., 2015). In addition, glaciers discharge freshwater and suspended loads that cause steep environmental gradients in salinity, temperature, and sedimentation rates (Svendsen et al., 2002). Others have shown that there is a reservoir of *Zetaproteobacteria* in coastal sediment habitats, where they may influence the coastal iron cycle (McBeth and Emerson, 2016). Further, it is well known that marine iron and sulfur cycles are tightly linked (Jorgensen et al., 2012). Regarding sulfur cycling, *Epsilonproteobacteria* are known to play important roles in the cycling of sulfidic compounds in various marine and terrestrial aquatic environments (Campbell et al., 2006). In Kongsfjorden sediments, sequences related to *Epsilonproteobacteria* (*Sulfurimonas* and *Sulfurovum* genera) comprised a significant proportion of sediment bacteria. In addition, *Sulfurovum* were more abundant in the shallow water sediment BJ36 (18.5% of the total bacterial sequences) than the deep water sediment BJ4 (9.7%). *Sulfurovum* can grow chemolithoautotrophically with hydrogen, sulfur and thiosulfate as an electron donor, and with oxygen, nitrate, thiosulfate, and sulfur as an electron acceptor using CO_2_ as a carbon source (Mino et al., 2014). Increasing seawater depth can influence the presence of anaerobic conditions in sediments. In this study, more *Desulfocapsa*-related sequences were detected in the deep-water sediment BJ4 (2.5%) than in shallow water sediments BJ36 (0.4%). The ability to disproportionate inorganic sulfur compounds has been documented for a number of anaerobic sulfate-reducing bacteria from the genus *Desulfocapsa* as well as the genus *Desulfobulbus* (Finster, 2008), which were both found to be abundant (1.1–8.8%) in Arctic and sub-Arctic sediments here. *Lutibacter-*related sequences that were represented by OTU1751 were unexpectedly abundant in the Kongsfjorden sediment BJ36 (7.4%), but were seldom detected in sediment BJ4. The OTU shared 94.1% sequence similarity to the microaerophilic and organotrophic *Lutibacter profundi* LP1^T^ isolated from an Arctic deep-sea hydrothermal vent system (Wissuwa et al., 2017). To date, all published *Lutibacter* strains have been isolated from marine environments (Wissuwa et al., 2017). Thus, further analyses are needed to understand the high abundance of *Lutibacter* in Kongsfjorden sediment BJ36, which is clearly influenced by glacial meltwater.

Nearly half of the archaeal sequences in the inner basin sediment BJ36 were closely related to the methanogenic archaeal class *Methanomicrobia*, and specifically the genus *Methanococcoides*, while this class was only 0.8% of the total archaeal sequences in the outer basin sediment BJ4. Considering that sediment BJ36 was located in the Kongsbreen glacial bay and was very close to the seashore, and thus also shallow seawaters (Zhu et al., 2014), the predominance of *Methanococcoides* in sediment BJ36 can be attributed to glacier meltwater and runoff waters carrying soils, which then delivers a large number of terrestrial microbial taxa into the fjord. A substantial number of methanogens, including Euryarchaeota *Methanomicrobia*, have been reported previously in tundra soils (Tveit et al., 2013) and polar desert soils (McCann et al., 2016) from the Kongsfjorden region. At the same time, it is worth mentioning that, as described above, *Zetaproteobacteria* were exclusively abundant in sediment BJ36. Zetaproteobacterial sequences have been reported to exist in methane seep sediments (Nunoura et al., 2012). In contrast, Euryarchaeota *Halobacteria* and thaumarchaeotal MBG-B were more abundant in the deep water sediment BJ4 than the shallow water sediment BJ36. *Halobacteria* require Na^+^ for growth, and can grow aerobically or anaerobically (DasSarma et al., 2012). *Halobacteria* dominance of archaeal communities has been reported in Shark Bay mat microbiomes (Wong et al., 2017). Thaumarchaeotal MBG-B, which is synonymous with the Deep-Sea Archaeal group (Spang et al., 2015), has been found in a wide range of marine sediments and hydrothermal vent samples (Knittel et al., 2005; Durbin and Teske, 2012; Jorgensen et al., 2012; Lauer et al., 2016), but appears to be primarily found in organic-rich, reducing sediments (Durbin and Teske, 2012). Glacial activity can have an effect on environmental gradients in salinity, temperature, sedimentation rates, and bottom sediment compositions (Svendsen et al., 2002), and thus has a great impact on sediments in the inner fjord. In addition, the deposited material in the glacial bay area is not compacted and frequently resuspended by iceberg scouring, sediment slides, and gravity flows (Wlodarska-Kowalczuk and Pearson, 2004). The strong environmental gradients in sedimentation and freshwater input likely induce large changes in community composition and abundances from the inner to the outer fjord (Hop et al., 2002). These environmental gradients likely also lead to the differences observed in microbial community structure between sediment BJ36 in the inner fjord and sediment BJ4 in the outer fjord.

Although similar archaeal and bacterial members were present in the shallow shelf sediment NEC5 and the deep basin sediment DBS1 in the northern Bering Sea, the abundances of some archaeal and bacterial populations differed significantly between the two samples. Sequences affiliated with *Thaumarchaeota* MG-I (represented by *Candidatus* Nitrosopumilus), were significantly more abundant in sediment DBS1 compared to NEC5. MG-I are one of the most abundant and cosmopolitan chemoautotrophs within the global dark ocean (Swan et al., 2014), and the distribution of MG-I in marine sediments can be linked to the availability of oxygen and nitrate in sediment porewaters which can serve as electron acceptors (Lauer et al., 2016). *Thaumarchaeota* MBG-B was also in higher abundance in sediment DBS1 compared to NEC5. In contrast, sequences affiliated with *Thaumarchaeota* Group C3 were much more abundant in sediment NEC5 than in DBS1. Regarding bacterial community differences, sequences affiliated with the *Alphaproteobacteria, Acidobacteria, Actinobacteria, Chloroflexi, Gemmatimonadetes*, and *Planctomycetes* were in much higher abundance in sediment DBS1, while *Gammaproteobacteria* and *Bacteroidetes* were significantly more abundant in sediment NEC5. In addition to water depth and the geographic location (**Table 1**), variation in salinity and nutrient concentrations of the overlying bottom water are considerably different in sediments NEC5 and DBS1 in the northern Bering Sea (Cooper et al., 2012), which potentially contributes to the observed differences in sediment microbial community composition.

In this study, archaeal and bacterial core microbiomes were observed among the four sediments. *Thaumarchaeota* Group C3-related *Archaea* were dominant in all four sediments (represented by OTU59, OTU73, OTU97, OTU145, and OTU170), indicating an important ecological role in both Arctic and sub-Arctic sediment communities. Members of Group C3 have been found across a wide range of terrestrial and marine environments, and they often dominate oceanic sediment surfaces and deep subsurface sediment archaeal communities (Hugoni et al., 2015; Na et al., 2015). Others have suggested that this archaeal group may be involved in acetate cycling within sulfate-reducing marine sediments (Na et al., 2015). Further, sequences affiliated with *Candidatus* Nitrosopumilus (represented by archaeal OTU21 and OTU275) comprised a substantial proportion (8.5–33.7%) of the *Archaea* in each sediment archaeal community in the northern Bering Sea, while they were in low abundance in Kongsfjorden sediments. *Candidatus* Nitrosopumilus is the major ammonia oxidizing archaea (AOA) taxonomic group (Pester et al., 2012). Interestingly, sequences related to ammonia-oxidizing bacteria (AOB), including the genera *Nitrosomonas, Nitrospina*, and *Nitrospira*, were seldom or not at all detected in the sediments sampled here. Thus, our results suggest that AOA may play a more important role in nitrogen cycling in the sediment of the northern Bering Sea.

Sequences affiliated with the genus *Desulfobulbus* (represented by bacterial OTU4057), which are sulfate and iron reducers (Holmes et al., 2004), were dominant in all sediments, indicating a predominance of sulfate-reducing *Bacteria* in both Arctic and sub-Arctic sediments. Further, sequences affiliated with the genus *Sulfurovum* (represented by bacterial OTU1750), were also found in four sediments, but were in significantly higher abundance in Kongsfjorden than in the northern Bering Sea sediments. In addition, *Sulfurimonas*-related sequences were also dominant in Kongsfjorden sediments but were seldom detected in the northern Bering Sea sediments. *Acidiferrobacter*-related sequences (represented by bacterial OTU2773) were detected among the four sediments, and were particularly abundant (4.5%) in the shallow shelf sediment NEC5 of the northern Bering Sea. The genus *Acidiferrobacter* is characterized as a facultatively anaerobic iron- and sulfur-oxidizer of the family *Ectothiorhodospiraceae* (Hallberg et al., 2011). In contrast, sequences related to sulfur-oxidizing bacteria were seldom found in the deep-sea sediment DBS1 of the northern Bering Sea. These observed differences in the diversity of sulfur-oxidizing bacteria in sediments suggest variation in sediment microbial community structure and bacterial ecological function between Kongsfjorden and the northern Bering Sea. Moreover, these differences point to ecological differentiation between the shallow shelf sediments and deep-sea sediments in the northern Bering Sea. The genus *Marinicella* within the *Gammaproteobacteria* (represented by bacterial OTU3421), was dominant in all four sediments. However, *Marinicella*-related sequences accounted for 9.6 and 6.5% of *Bacteria* in shelf sediment NEC5 and deep basin sediment DBS1 of the northern Bering Sea, respectively, which contrasts with their lower abundance (<1.5% of bacterial sequences) in Kongsfjorden sediment communities. The genus *Marinicella* contains two recognized species, *M. litoralis* and *M. pacifica*, which were both isolated from seawater in the Pacific region (Romanenko et al., 2010; Wang et al., 2016). This result is in agreement with sediment NEC5 from the Bering shelf being more directly influenced by waters with Pacific origin compared to the deep water sediment DBS1.

## Conclusion

Our use of 454 pyrosequencing technology has greatly expanded our knowledge of the prokaryotic community composition in sediments of the Arctic Kongsfjorden and sub-Arctic northern Bering Sea, and has revealed a vast archaeal and bacterial diversity in contrast to previous investigations based on Sanger sequencing-based approaches. Here, we also report archaeal community compositions for the first time in sediments of the northern Bering Sea. Dominance of the *Thaumarchaeota* Group C3, *Desulfobulbus* and *Marinicella* were observed among the four sediments sampled here. However, further research is required to understand the ecological role of *Thaumarchaeota* Group C3 and *Marinicella* in marine sediments. Variation in prokaryotic community composition was detected among these sediments, suggesting varying influences of geographic location, hydrographic characteristics and local geochemical features on these sediment microbial communities. Further research is required to fully understand the correlations between sediment prokaryotic community composition, physicochemical parameters and sediment types. AOA were found to be much more abundant than AOB in sediments, especially in the northern Bering Sea sediments, which suggests a more significant role of *Archaea* in nitrogen cycling in pan-arctic marine sediments. Sequences affiliated with the sulfur-oxidizing genera *Acidiferrobacter, Sulfurimonas*, and *Sulfurovum* and the sulfate-reducing genus *Desulfobulbus* were detected in all sediments, implicating a sulfur cycling role of *Bacteria* in those sediments. Further research is required to fully understand the ecological roles of *Archaea* and *Bacteria* in carbon, nitrogen, and sulfur cycling in these environments.

## Author Contributions

This study was conceived, designed, and performed by Y-XZ. Y-XZ conducted the data analysis, interpretation, and manuscript preparation with assistance from YY, H-RL, and WL. Y-XZ wrote the manuscript. Y-XZ, YY, H-RL, and WL have reviewed the final version for submission for publication, and agree to be accountable for all aspects of the work in ensuring that questions related to the accuracy or integrity of any part of the work are appropriately investigated and resolved.

## Conflict of Interest Statement

The authors declare that the research was conducted in the absence of any commercial or financial relationships that could be construed as a potential conflict of interest.

## Footnotes

1 http://drive5.com/usearch/

2 http://www.arb-silva.de

3 http://www.mothur.org
