# Supplementary material for: Prokaryotic Community Composition in Arctic Kongsfjorden and Sub-Arctic Northern Bering Sea Sediments As Revealed by 454 Pyrosequencing
Source: Front Microbiol. 2017 Dec 12;8:2498. doi: 10.3389/fmicb.2017.02498 (PMC5732994; doi:10.3389/fmicb.2017.02498)
Supplement: Supplementary file 1 [file Data_Sheet_1.DOCX]

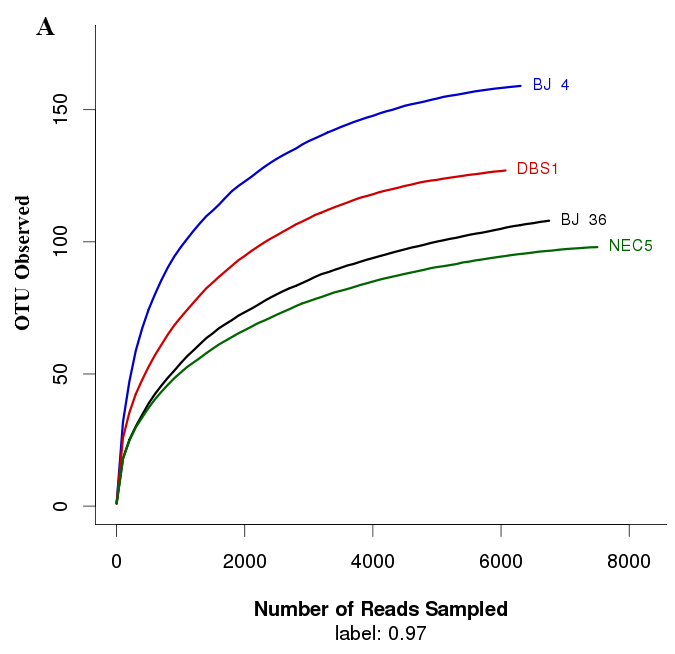

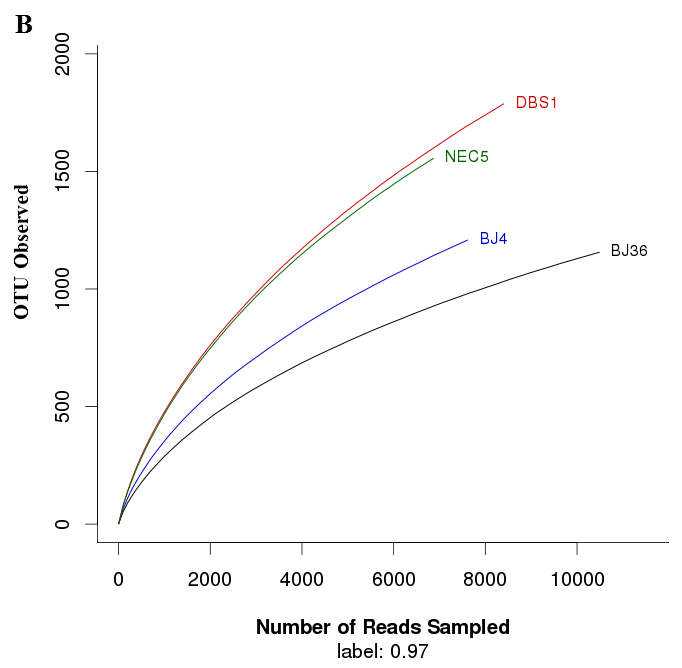


**Figure S1. Rarefaction curves for (A) *Archaea* and (B) *Bacteria* from sediment samples.** Operational taxonomic units (OTUs) in this analysis were defined at 97% sequence identity.


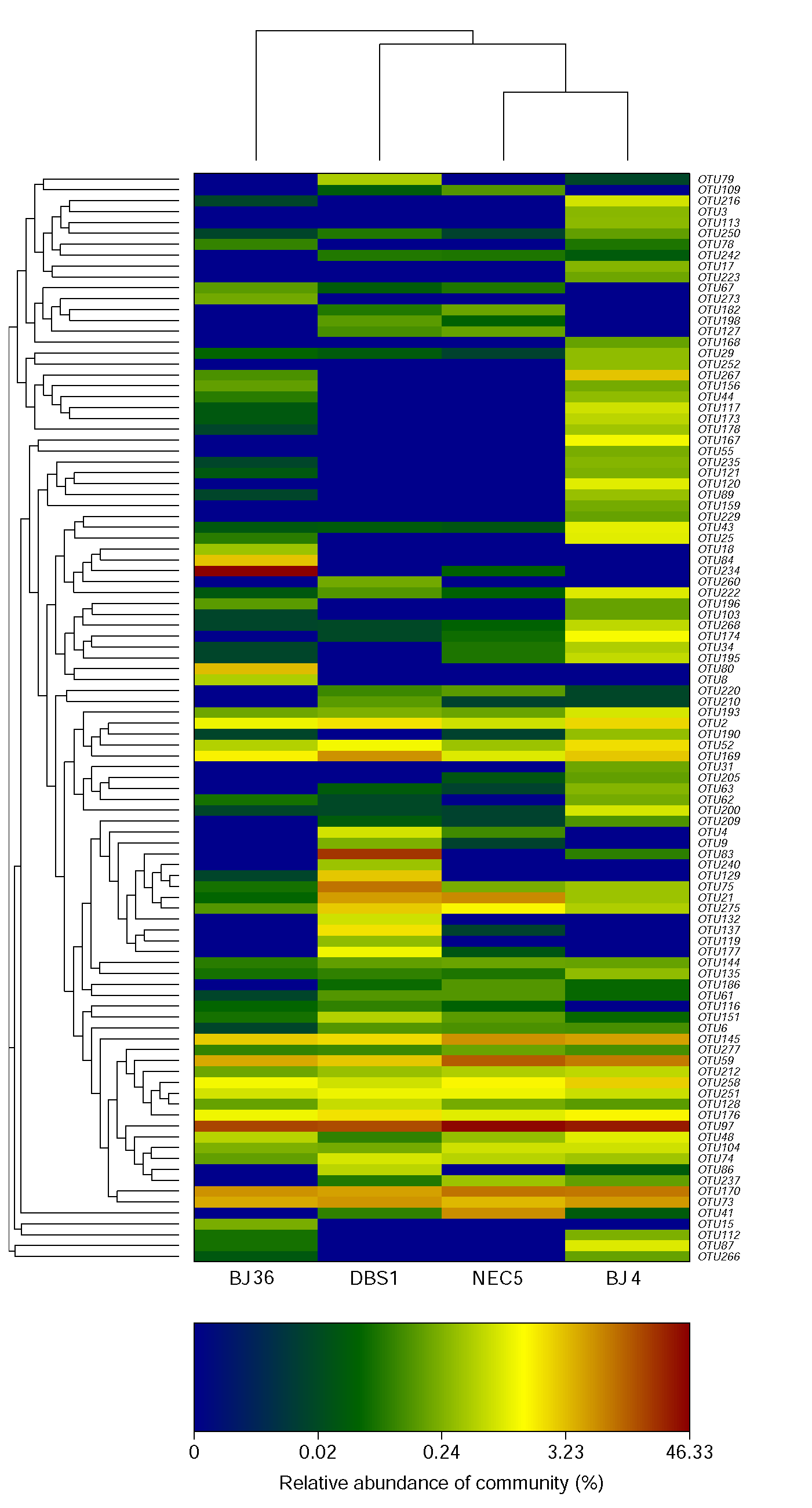


**Figure S2.** **A heatmap diagram visualizing the 100 most abundant archaeal OTUs among the four sediment samples.**


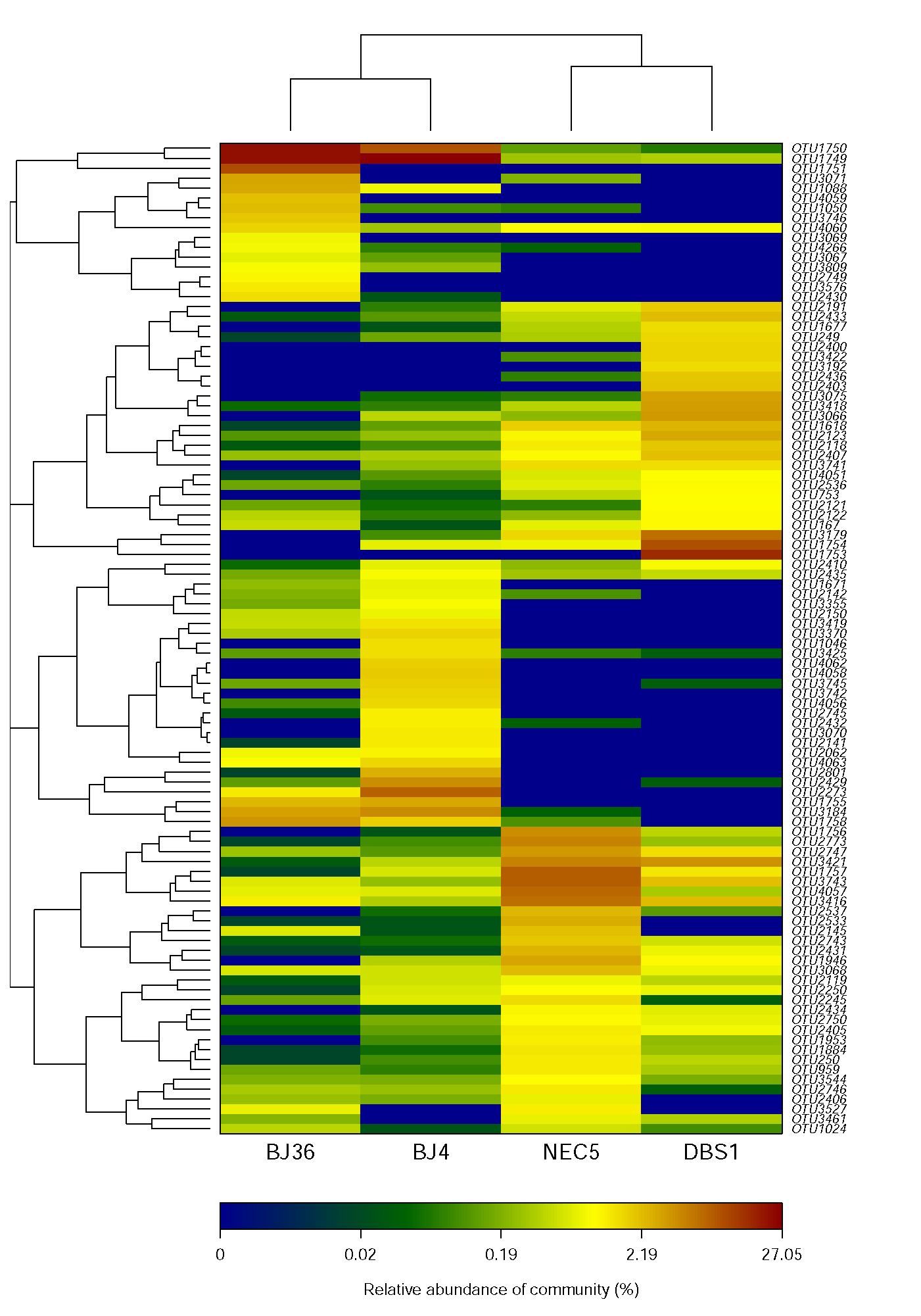


**Figure S3.** **A heatmap diagram visualizing the 100　most abundant bacterial OTUs among the four sediment samples.**
